# Development of FAPI Tetramers to Improve Tumor Uptake and Efficacy of FAPI Radioligand Therapy

**DOI:** 10.2967/jnumed.123.265599

**Published:** 2023-09

**Authors:** Yizhen Pang, Liang Zhao, Jianyang Fang, Jianhao Chen, Lingxin Meng, Long Sun, Hua Wu, Zhide Guo, Qin Lin, Haojun Chen

**Affiliations:** 1 Department of Nuclear Medicine and Minnan PET Center, Xiamen Key Laboratory of Radiopharmaceuticals, The First Affiliated Hospital of Xiamen University, School of Medicine, Xiamen University, Xiamen, China;; 2 Department of Radiation Oncology, Xiamen Cancer Center, Xiamen Key Laboratory of Radiation Oncology, First Affiliated Hospital of Xiamen University, School of Medicine, Xiamen University, Xiamen, China;; 3 Departments of Diagnostic Radiology, Surgery, Chemical and Biomolecular Engineering, and Biomedical Engineering, Yong Loo Lin School of Medicine and Faculty of Engineering, National University of Singapore, Singapore;; 4 Clinical Imaging Research Centre, Centre for Translational Medicine, Yong Loo Lin School of Medicine, National University of Singapore, Singapore; and; 5 State Key Laboratory of Molecular Vaccinology and Molecular Diagnostics and Center for Molecular Imaging and Translational Medicine, School of Public Health, Xiamen University, Xiamen, China

**Keywords:** fibroblast activation protein, cancer-associated fibroblasts, tetramer, dimer, radioligand therapy

## Abstract

Radiolabeled fibroblast activation protein (FAP) inhibitors (FAPIs) have shown promise as cancer diagnostic agents; however, the relatively short tumor retention of FAPIs may limit their application in radioligand therapy. In this paper, we report the design, synthesis, and evaluation of a FAPI tetramer. The aim of the study was to evaluate the tumor-targeting characteristics of radiolabeled FAPI multimers in vitro and in vivo, thereby providing information for the design of FAP-targeted radiopharmaceuticals based on the polyvalency principle. **Methods:** FAPI tetramers were synthesized on the basis of FAPI-46 and radiolabeled with ^68^Ga, ^64^Cu, and ^177^Lu. In vitro FAP-binding characteristics were identified using a competitive cell-binding experiment. To evaluate their pharmacokinetics, small-animal PET, SPECT, and ex vivo biodistribution analyses were performed on HT-1080-FAP and U87MG tumor–bearing mice. In addition, the 2 tumor xenografts received radioligand therapy with ^177^Lu-DOTA-4P(FAPI)_4_, and the antitumor efficacy of the ^177^Lu-FAPI tetramer was evaluated and compared with that of the ^177^Lu-FAPI dimer and monomer. **Results:** ^68^Ga-DOTA-4P(FAPI)_4_ and ^177^Lu-DOTA-4P(FAPI)_4_ were highly stable in phosphate-buffered saline and fetal bovine serum. The FAPI tetramer exhibited high FAP-binding affinity and specificity both in vitro and in vivo. ^68^Ga-, ^64^Cu-, and ^177^Lu-labeled FAPI tetramers exhibited higher tumor uptake, longer tumor retention, and slower clearance than FAPI dimers and FAPI-46 in HT-1080-FAP tumors. The uptake (percentage injected dose per gram) of ^177^Lu-DOTA-4P(FAPI)_4_, ^177^Lu-DOTA-2P(FAPI)_2_, and ^177^Lu-FAPI-46 in HT-1080-FAP tumors at 24 h was 21.4 ± 1.7, 17.1 ± 3.9, and 3.4 ± 0.7, respectively. Moreover, ^68^Ga-DOTA-4P(FAPI)_4_ uptake in U87MG tumors was approximately 2-fold the uptake of ^68^Ga-DOTA-2P(FAPI)_2_ (SUV_mean_, 0.72 ± 0.02 vs. 0.42 ± 0.03, *P* < 0.001) and more than 4-fold the uptake of ^68^Ga-FAPI-46 (0.16 ± 0.01, *P* < 0.001). In the radioligand therapy study, remarkable tumor suppression was observed with the ^177^Lu-FAPI tetramer in both HT-1080-FAP and U87MG tumor–bearing mice. **Conclusion:** The satisfactory FAP-binding affinity and specificity, as well as the favorable in vivo pharmacokinetics of the FAPI tetramer, make it a promising radiopharmaceutical for theranostic applications. Improved tumor uptake and prolonged retention of the ^177^Lu-FAPI tetramer resulted in excellent characteristics for FAPI imaging and radioligand therapy.

## Visual Abstract

**Figure:**
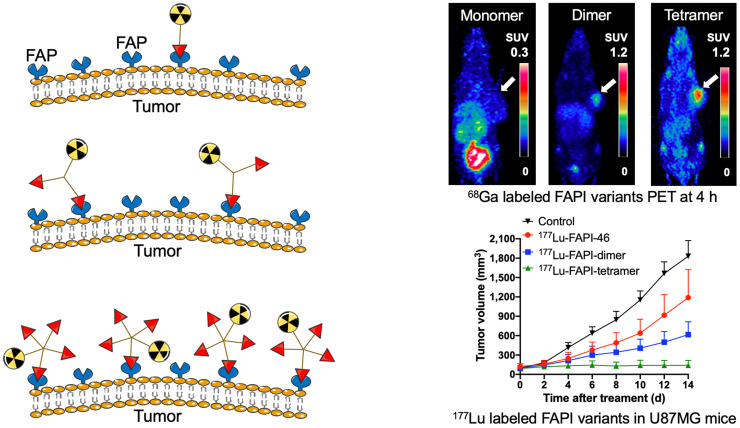

Cancer-associated fibroblasts, which are major components of the tumor stroma in many epithelial carcinomas, play a pivotal role in tumor growth, tissue remodeling, and immune evasion (1). Fibroblast activation protein (FAP), a type II transmembrane glycoprotein, is overexpressed in cancer-associated fibroblasts but expressed at low levels in normal fibroblasts (2). Therefore, FAP is considered a promising target for tumor imaging and therapy.

Several quinoline-based FAP inhibitors (FAPIs) have been developed (3–6). ^68^Ga-FAPI-46 appeared to be the most promising derivatives in the series, providing a favorable tumor-to-background ratio and good tumor accumulation (3*,*^7^). However, their relatively short tumor retention may limit the use of radiolabeled FAPIs for radioligand therapy (8*,*^9^). Various chemical optimization strategies for theranostic applications, including cyclization, multimerization, and albumin binding, reportedly improve tumor uptake and prolong tumor retention of these radioligands (9–11).

In our previous study, a dimeric FAPI molecule, DOTA-2P(FAPI)_2_, was designed and synthesized (12). Preclinical and clinical PET studies have demonstrated that ^68^Ga-DOTA-2P(FAPI)_2_ exhibits significantly higher tumor uptake and longer retention than ^68^Ga-FAPI-46 (12). Similar results were obtained for other FAPI dimers, including DOTAGA, (SA.FAPi)_2_, and BiOncoFAP (13*,*^14^). Therefore, polyvalency may be an effective strategy for developing FAP-targeted radiopharmaceuticals with higher tumor uptake because of their increased FAP-recognition ability. Moreover, FAP-targeted radioligand therapy could be more effective if further improvements in tumor retention and absolute uptake are achieved.

In this paper, we report the design, synthesis, and preclinical evaluation of a tetrameric FAPI molecule based on the polyvalency principle. It was constructed on the FAPI-46 motif with 4 diethylene glycol (mini–polyethylene glycol [PEG]) spacers between the 4 FAPI motifs, denoted as 4P(FAPI)_4_. This FAPI tetramer was conjugated to the chelator DOTA or NOTA and labeled with ^68^Ga or ^64^Cu for PET imaging. It was also labeled with ^177^Lu for radioligand therapy applications. This study aimed to investigate the tumor-targeting potential of FAPI tetramers in vitro and in vivo and whether this form is more effective than its monomeric and dimeric analogs.

## MATERIALS AND METHODS

### Chemistry and Radiolabeling

Details of the reagents, chemicals, synthesis route, radiochemistry, and quality control of the FAPI tetramer are described in the supplemental materials (available at http://jnm.snmjournals.org) (12). For ^68^Ga labeling, approximately 25.4 nmol of FAPI-46, DOTA-2P(FAPI)_2_, or DOTA-4P(FAPI)_4_ were dissolved in 1 mL of NaAc (0.25 M in water) and added to 4 mL of ^68^GaCl_3_ solution (1.3 GBq in 0.05 M HCl). The mixture was incubated at 95°C for 10 min. For ^177^Lu labeling, each of the aforementioned precursors was dissolved in 1 mL of NaAc (0.25 M in water) and added to 4 mL of ^177^LuCl_3_ solution (740 MBq in 0.05 M HCl). The mixture was incubated at 95°C for 30 min. To allow stable complexation of ^64^Cu, the DOTA group of the FAPI dimer or tetramer was replaced with NOTA. Approximately 26.7 nmol of NOTA-2P(FAPI)_2_ or NOTA-4P(FAPI)_4_ were diluted with 450 μL of NaAc (0.5 M) and incubated with 50 μL of ^64^CuCl_2_ (740 MBq in 0.01 M HCl) at 90°C for 20 min. All 3 products were purified using a C18 Plus Short Cartridge (WAT020515; Waters Corp.). Radio–high-performance liquid chromatography was used for quality control.

### Cell Culture and in Vitro Evaluation

A human fibrosarcoma cell line was stably transfected with FAP (HT-1080-FAP) and cultured, as previously described (15). A human glioblastoma cell line (U87MG, from the Chinese National Infrastructure of Cell Line Resource) was cultured in Dulbecco modified Eagle medium (Thermo Fischer Scientific) supplemented with 10% fetal bovine serum (Thermo Fisher Scientific). HT-1080-FAP cells were seeded in 24-well plates using RPMI 1640 medium with 10% fetal bovine serum, and the medium was replaced with fresh medium without fetal bovine serum when the cells reached 80%–90% density. ^68^Ga-FAPI-46, ^68^Ga-DOTA-2P(FAPI)_2_, ^68^Ga-DOTA-4P(FAPI)_4_, or ^68^Ga-DOTA-4P(FAPI)_4_ with 11.3 nmol of unlabeled FAPI-46 (for the blocking experiment) were added to the 24-well plates and incubated for 60, 90, and 120 min. For the competitive cell-binding assay, a gradient concentration (5.6 × 10^−13^ to 10^−5^ M) of FAPI-46, DOTA-2P(FAPI)_2_, or DOTA-4P(FAPI)_4_ was added to the cells and incubated with ^68^Ga-FAPI-46 for 60 min. Similarly, a gradient concentration (5.4 × 10^−13^ to 10^−5^ M) of NOTA-2P(FAPI)_2_ or NOTA-4P(FAPI)_4_ was added to the cells and incubated with ^68^Ga-FAPI-46 for 60 min. After each step, the cells were washed twice with phosphate-buffered saline (1 mL) and lysed with 1 M NaOH (0.5 mL). The radioactivity (counts per minute) was determined with a γ-counter (WIZARD^2^ 2480; PerkinElmer Inc.). All experiments were independently repeated 3 times. The 50% inhibitory concentrations were determined by fitting a nonlinear regression model to the data using Prism software, version 8 (GraphPad Software Inc.).

### Preparation of Cell Line–Derived Xenograft Models

All animal experimental procedures were approved by the Animal Care and Ethics Committee of Xiamen University and performed in accordance with the Guidelines for the Care and Use of Animals of the Xiamen University Laboratory Animal Center. For in vivo experiments, 6-wk-old BALB/c nude mice (Beijing Vital River Laboratory Animal Technology Co., Ltd.) were subcutaneously inoculated with HT-1080-FAP or U87 cells (5 × 10^6^ in 100 μL of phosphate-buffered saline) in the right shoulder.

### Small-Animal PET and SPECT Studies

Dynamic PET, static PET (with or without competition), and SPECT scans with radiolabeled monomeric, dimeric, and tetrameric FAPIs were performed on HT-1080-FAP tumor–bearing mice for pharmacokinetic evaluation. Additionally, static PET with ^68^Ga-labeled monomeric, dimeric, and tetrameric FAPIs was performed and compared in U87MG tumor–bearing mice.

Approximately 7.4 MBq of ^68^Ga-DOTA-4P(FAPI)_4_ was intravenously injected into HT-1080-FAP tumor–bearing mice (*n* = 3) for the 60-min dynamic PET. For multiple-time-point static PET (0.5, 1, 2, and 4 h after injection), 7.4 MBq of ^68^Ga-FAPI-46, ^68^Ga-DOTA-2P(FAPI)_2_, or ^68^Ga-DOTA-4P(FAPI)_4_ were injected into HT-1080-FAP and U87MG tumor–bearing mice (3/group). For longer-term observation, HT-1080-FAP tumor–bearing mice were intravenously injected with 7.4 MBq of ^64^Cu-NOTA-2P(FAPI)_2_ or ^64^Cu-NOTA-4P(FAPI)_4_ (3/group). For the in vivo blocking experiment, PET imaging was performed 1 h after the simultaneous administration of 30 nmol of unlabeled FAPI-46 and 7.4 MBq of ^68^Ga-DOTA-4P(FAPI)_4_.

SPECT scans were conducted from 1 to 96 h with 18.5 MBq of ^177^Lu-FAPI-46, ^177^Lu-DOTA-2P(FAPI)_2_, or ^177^Lu-DOTA-4P(FAPI)_4_ in HT-1080-FAP tumor–bearing mice (3/group). Details of the machine settings, dynamic and static PET imaging procedures, static SPECT imaging procedures, imaging acquisition, and image reconstruction are presented in the supplemental materials.

### Biodistribution Study

Three groups of HT-1080-FAP mice were injected with 0.74 MBq of ^177^Lu-FAPI-46, ^177^Lu-DOTA-2P(FAPI)_2_, or ^177^Lu-DOTA-4P(FAPI)_4_ and were euthanized at different time points (24–48 h for monomers and 24–96 h for dimers and tetramers, 3/group). Blood, tumor, muscle, and major organs were weighed and measured using a γ-counter (WIZARD^2^ 2480). Data were normalized to the percentage injected dose per gram (%ID/g) using 1% of total counts.

### FAP-Targeted Radioligand Therapy

When the tumor volume reached approximately 100 mm^3^, the mice were randomized into 4 groups for radioligand therapy with ^177^Lu-labeled monomeric, dimeric, and tetrameric FAPIs (6/group): group A, saline; group B, 29.6 MBq of ^177^Lu-FAPI-46; group C, 29.6 MBq of ^177^Lu-DOTA-2P(FAPI)_2_; and group D, 29.6 MBq of ^177^Lu-DOTA-4P(FAPI)_4_. The frequency of administering ^177^Lu radiopharmaceuticals to U87MG mice was based on the administration frequency used in our previous study on hepatocellular carcinoma patient–derived xenograft tumor models, which showed a significant reduction in tumor uptake after 72 h after injection (15). HT-1080-FAP, a FAP-transfected tumor xenograft with much higher levels of FAP expression than U87MG, was also used in this study. Therefore, the frequency of administration was higher in U87MG tumor–bearing mice (every 72 h, 3 doses in total) than in the HT-1080-FAP models (a single dose). Weight and tumor volume were monitored every 2 d, and the mice were euthanized when the average tumor volume exceeded 1,500 mm^3^, when the tumor was ulcerated, or when weight loss was more than 20%. To further assess radioligand therapy–related toxicity effects, the main organs were collected from the ^177^Lu-DOTA-4P(FAPI)_4_ group on day 22 after hematoxylin and eosin staining (16).

### Statistics

Quantitative data are expressed as mean ± SD. Statistical analyses were performed using SPSS Statistics for Microsoft Windows, version 22.0 (IBM Corp.). The Student *t* test was used to determine differences between 2 groups, and 1-way ANOVA was used to compare differences among multiple groups. Statistical significance was set at a *P* value of less than 0.05.

## RESULTS

### Synthesis and Radiolabeling

**FIGURE 1.**
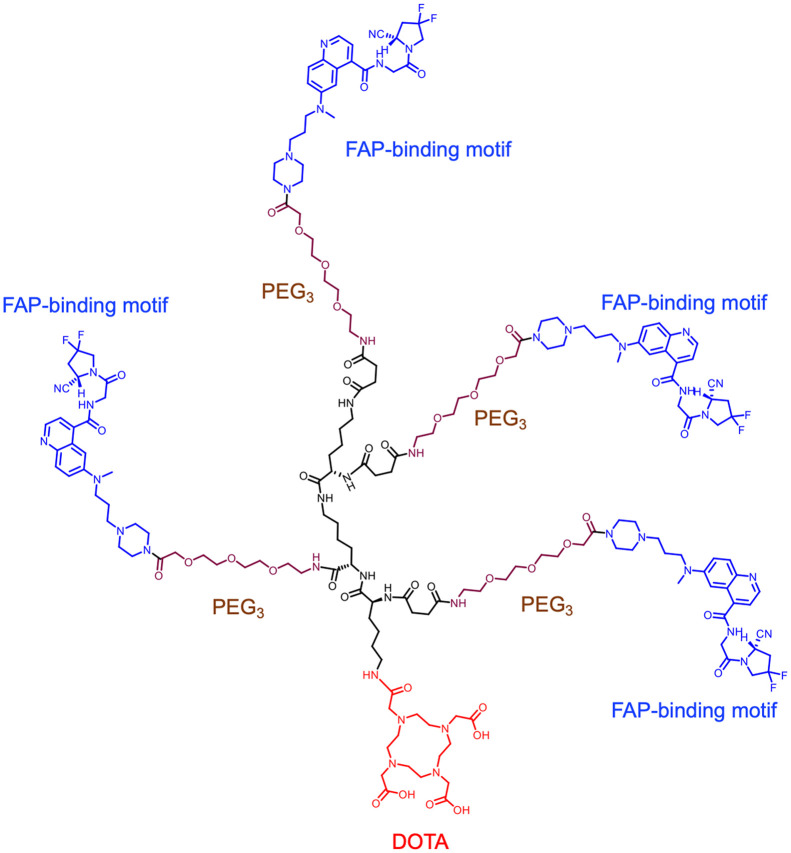
Chemical structure of FAPI tetramer DOTA-4P(FAPI)_4_.

Tetrameric FAPIs containing 4 PEG_3_ groups and the chelator DOTA or NOTA were synthesized (Fig. 1; Supplemental Figs. 1 and 2). ^68^Ga, ^64^Cu, and ^177^Lu were labeled in more than 90% yield with radiochemical purity of more than 95%. The specific activity was 32.0–36.4 GBq/μmol for ^68^Ga-labeled FAPI variants, 22.2–24.9 GBq/μmol for ^64^Cu-labeled FAPI variants, and 23.3–26.2 GBq/μmol for ^177^Lu-labeled FAPI variants.

Regarding in vitro stability, neither significant demetallation nor free radioactivity was observed 4 h (^68^Ga-DOTA-4P[FAPI]_4_) or 24 h (^177^Lu-DOTA-4P[FAPI]_4_) after incubation in phosphate-buffered saline and fetal bovine serum via radio–high-performance liquid chromatography analysis, demonstrating the high stability of the products (Supplemental Fig. 3).

### Cell-Binding Assay

**FIGURE 2.**
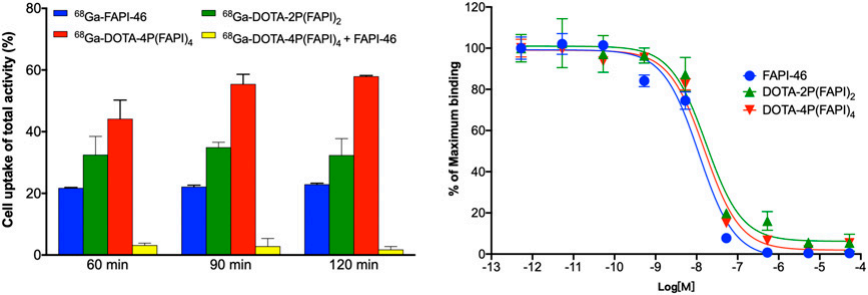
Cell uptake–blocking experiments and competitive cell-binding assay of FAPI-46, DOTA-2P(FAPI)_2_, and DOTA-4P(FAPI)_4_ using HT-1080-FAP cells.

In the cell uptake and blocking experiments, ^68^Ga-DOTA-4P(FAPI)_4_ yielded significantly higher uptake than ^68^Ga-DOTA-2P(FAPI)_2_ and ^68^Ga-FAPI-46 (57.98% ± 0.27% vs. 32.40% ± 5.36% vs. 22.93% ± 0.33% at 120 min). Moreover, unlabeled FAPI-46 significantly blocked ^68^Ga-DOTA-4P(FAPI)_4_ binding to FAP (57.98% ± 0.27% vs. 1.79% ± 0.97% at 120 min, a 97% reduction), confirming the FAP-targeting specificity of the FAPI tetramer (Fig. 2).

All 3 FAPI molecules (monomers, dimers, and tetramers) inhibited the binding of ^68^Ga-FAPI-46 to FAP-positive HT-1080-FAP cells in a dose-dependent manner. The 50% inhibitory concentrations for FAPI-46, DOTA-2P(FAPI)_2_, and DOTA-4P(FAPI)_4_ were comparable (11.38, 17.04, and 15.56 nM, respectively), indicating that tetramerization and dimerization have minimal effect on the FAP-binding affinity (Fig. 2). Additionally, the 50% inhibitory concentrations for NOTA-2P(FAPI)_2_ and NOTA-4P(FAPI)_4_ were also comparable (25.18 and 16.27 nM) (Supplemental Fig. 4).

### Small-Animal PET Imaging of HT-1080-FAP Tumors

**FIGURE 3.**
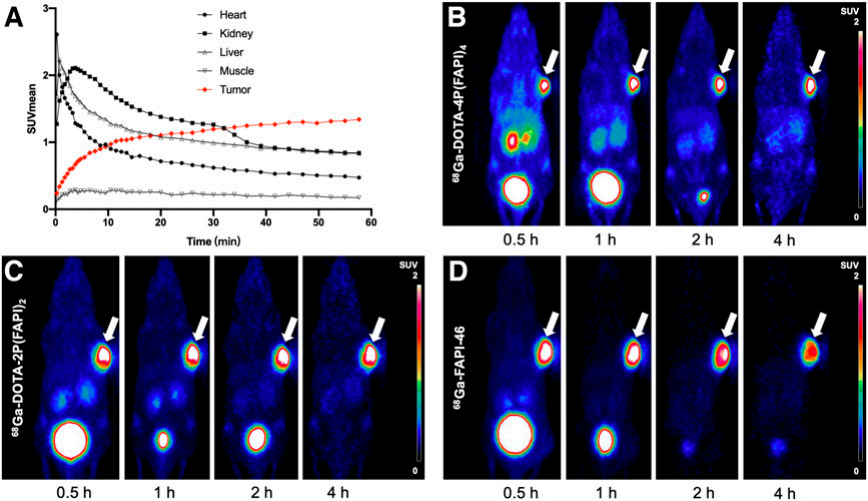
(A) Dynamic time–activity curves of ^68^Ga-DOTA-4P(FAPI)_4_ in heart, liver, kidney, muscle, and tumor of HT-1080-FAP tumor–bearing mice. (B–D) Representative PET images of ^68^Ga-DOTA-4P(FAPI)_4_, ^68^Ga-DOTA-2P(FAPI)_2_, and ^68^Ga-FAPI-46 in HT-1080-FAP tumor–bearing mice.

To comprehensively evaluate the in vivo pharmacokinetics of ^68^Ga-DOTA-4P(FAPI)_4_, a 60-min dynamic PET scan was performed on HT-1080-FAP tumor–bearing mice. As illustrated in Figure 3A, ^68^Ga-DOTA-4P(FAPI)_4_ was rapidly taken up by the tumor, and the uptake increased from 10 to 60 min after injection. In contrast, the radiotracer uptake rapidly declined over the same period in the heart, kidneys, and liver. Additional late-time-point static scans performed on tumor-bearing mice revealed that tumor uptake remained constant up to 4 h after injection (Fig. 3B). Moreover, ^68^Ga-DOTA-4P(FAPI)_4_ was eliminated predominantly through the kidneys and bladder, resulting in low background activity and favorable tumor-to-background ratios, especially at later time points. Similar tumor uptake and retention were observed for ^68^Ga-DOTA-2P(FAPI)_2_ (Fig. 3C); however, a significant decrease in tumor uptake over time was observed on ^68^Ga-FAPI-46 PET (Fig. 3D).

**FIGURE 4.**
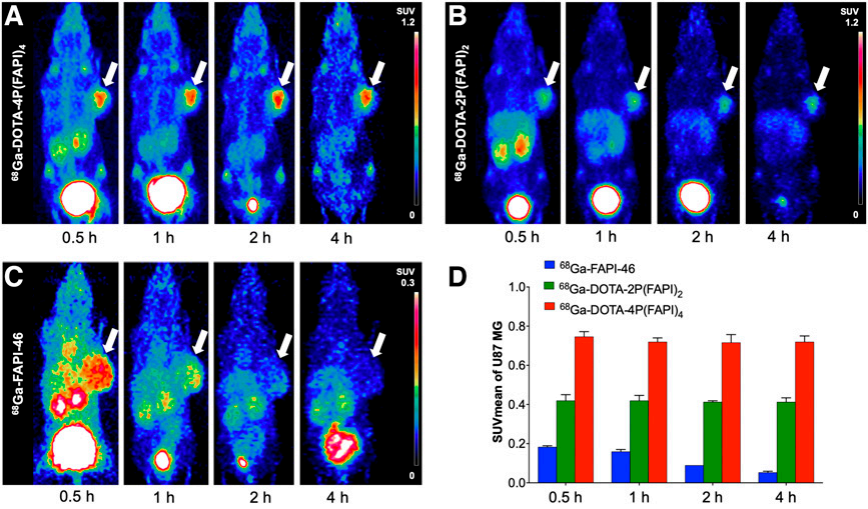
(A–C) Representative PET images of ^68^Ga-DOTA-4P(FAPI)_4_ (bar, SUV_mean_, 0–1.2) (A), ^68^Ga-DOTA-2P(FAPI)_2_ (bar, SUV_mean_, 0–1.2) (B), and ^68^Ga-FAPI-46 (bar, SUV_mean_, 0–0.3 because of low uptake) (C) in U87MG tumor–bearing mice. Arrows point toward tumor. (D) Tumor uptake of 3 radiotracers in U87MG tumors at 0.5–4 h after injection.

In terms of semiquantitative analysis, no significant difference was observed regarding tumor uptake among the 3 radiotracers at 1 h after injection, whereas uptake of ^68^Ga-DOTA-4P(FAPI)_4_ was significantly higher than that of FAPI dimer (SUV_mean_, 1.99 ± 0.09 vs. 1.71 ± 0.10, *P* = 0.018) and monomer (1.20 ± 0.07, *P* < 0.001) in HT-1080-FAP tumors at 4 h after injection (Supplemental Fig. 5). The difference in tumor uptake among the 3 radiotracers was more notable in U87MG tumor–bearing mice. As illustrated in Figure 4, the ^68^Ga-DOTA-4P(FAPI)_4_ uptake in U87MG tumors (1 h after injection) was approximately 2-fold higher than the ^68^Ga-DOTA-2P(FAPI)_2_ uptake (SUV_mean_, 0.72 ± 0.02 vs. 0.42 ± 0.03, *P* < 0.001) and more than 4-fold higher than the ^68^Ga-FAPI-46 uptake (0.16 ± 0.01, *P* < 0.001). In addition, washout of ^68^Ga-DOTA-4P(FAPI)_4_ and ^68^Ga-DOTA-2P(FAPI)_2_ from the U87MG tumor during the experimental time span was minimal, whereas a significantly decreased tumor uptake of ^68^Ga-FAPI-46 was observed.

Target specificity was evaluated using an in vivo blocking assay. Coinjection with an excess of unlabeled FAPI-46 successfully blocked tumor uptake at 1 h after injection (SUV_mean_ without blocking, 1.87 ± 0.08, vs. SUV_mean_ with blocking, 0.16 ± 0.03; 92% reduction in tumor uptake), demonstrating that the uptake of the major fraction of ^68^Ga-DOTA-4P(FAPI)_4_ in tumors was FAP-mediated (Supplemental Fig. 6).

**FIGURE 5.**
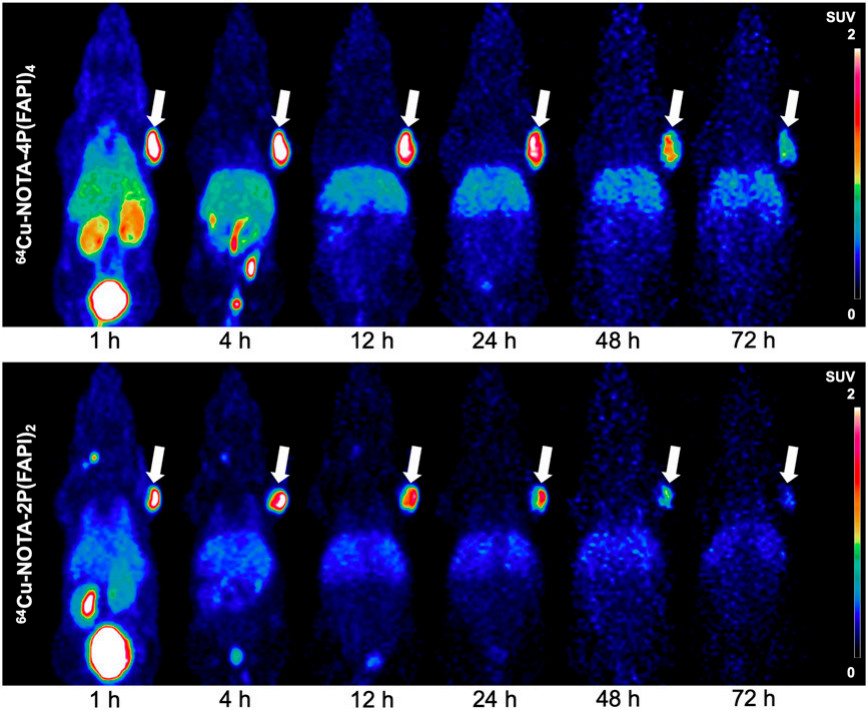
Representative PET imaging of ^64^Cu-NOTA-4P(FAPI)_4_ and ^64^Cu-NOTA-2P(FAPI)_2_ in HT-1080-FAP tumor–bearing mice. Arrows point toward tumor.

To observe the entire process of tracer accumulation and washout from the tumor tissue, a radionuclide with a longer half-life (12.7 h, ^64^Cu) was used to label the FAPI tetramers and dimers. During multiple-time-point static PET imaging, the uptake of ^64^Cu-NOTA-4P(FAPI)_4_ in HT-1080 FAP tumors was higher than that of ^64^Cu-NOTA-2P(FAPI)_2_ at all examined time points, and ^64^Cu-NOTA-4P(FAPI)_4_ washout was slightly slower than ^64^Cu-NOTA-2P(FAPI)_2_ washout during the experimental period (Fig. 5). Uptake of ^64^Cu-NOTA-4P(FAPI)_4_ in the kidney and liver was also higher than that of ^64^Cu-NOTA-2P(FAPI)_2_, whereas uptake in other nontarget organs was similar for both radiotracers. A detailed semiquantitative analysis of ^64^Cu-NOTA-4P(FAPI)_4_ uptake in the tumor and main organs is presented in Supplemental Figure 7.

### SPECT Imaging and Biodistribution of ^177^Lu-Labeled FAPI Tetramer in HT-1080-FAP Tumors

**FIGURE 6.**
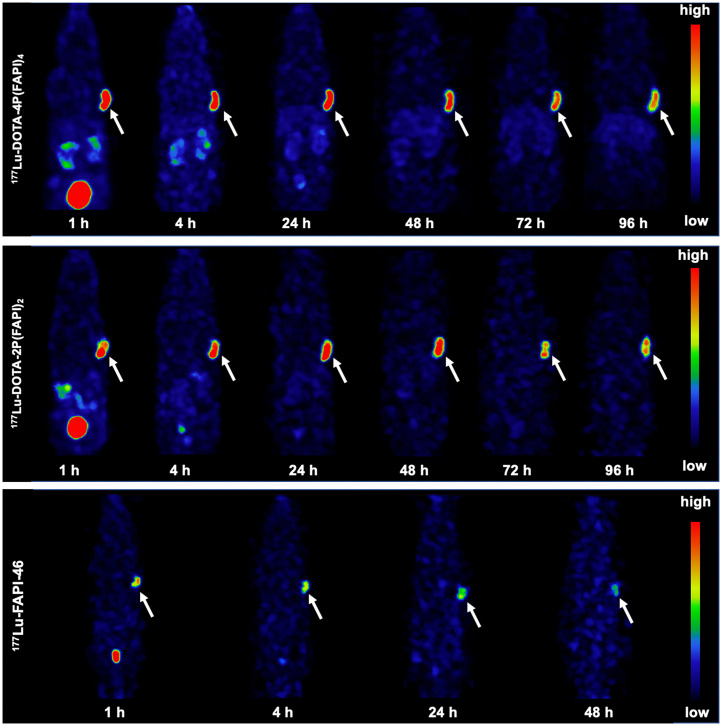
Representative SPECT images of ^177^Lu-DOTA-4P(FAPI)_4_, ^177^Lu-DOTA-2P(FAPI)_2_, and ^177^Lu-FAPI-46 in HT-1080-FAP tumor–bearing mice. Arrows point toward tumor.

Whole-body SPECT imaging and biodistribution studies were performed to further explore the in vivo characteristics of the ^177^Lu-labeled FAPI tetramer. Representative SPECT images of the FAPI tetramer, dimer, and monomer are presented in Figure 6 (3/group), and the ex vivo biodistribution data of the 3 tracers are presented in Supplemental Figure 8 (3 per group). Similar to the observation with ^64^Cu-labeled analogs, HT-1080-FAP tumors clearly contained ^177^Lu-labeled dimer and tetramer at all time points examined (Fig. 6). The uptake of ^177^Lu-DOTA-4P(FAPI)_4_ reached 21.4 ± 1.7 %ID/g 24 h after injection, with relatively slow tumor clearance (19.2 ± 0.6 %ID/g, 18.8 ± 2.1 %ID/g, and 14.8 ± 0.9 %ID/g at 48, 72, and 96 h, respectively). The tumor uptake of ^177^Lu-DOTA-2P(FAPI)_2_ was 17.1 ± 3.9 %ID/g 24 h after injection, which was slightly lower than that of ^177^Lu-DOTA-4P(FAPI)_4_. Tumor washout of the FAPI dimer was faster than that of the tetramer, with uptake values of 18.8 ± 4.1 %ID/g, 13.8 ± 2.6 %ID/g, and 13.1 ± 0.7 %ID/g at 48, 72, and 96 h, respectively. Unsurprisingly, the tumor uptake of ^177^Lu-FAPI-46 was significantly lower than that of ^177^Lu-DOTA-4P(FAPI)_4_ 24 h after injection (3.4 ± 0.7 %ID/g, *P* < 0.001). Because ^177^Lu-FAPI-46 was rapidly cleared from the blood and exhibited extremely low accumulation in the tumor 48 h after injection (2.0 ± 0.4 %ID/g), no further scans were performed for this radiotracer.

Similar to that of ^64^Cu-labeled analogs, the uptake of ^177^Lu-DOTA-4P(FAPI)_4_ in certain nontarget organs 48 h after injection was significantly higher than that of ^177^Lu-DOTA-2P(FAPI)_2_ and ^177^Lu-FAPI-46 (kidney: 6.6 ± 0.2 %ID/g vs. 2.9 ± 1.5 %ID/g and 0.4 ± 0.01 %ID/g; liver: 6.3 ± 0.5 %ID/g vs. 2.6 ± 0.9 %ID/g and 0.5 ± 0.02 %ID/g; and spleen: 5.1 ± 0.7 %ID/g vs. 2.0 ± 0.8 %ID/g and 0.2 ± 0.04 %ID/g).

### FAP-Targeted Radioligand Therapy with ^177^Lu-FAPI Tetramer

**FIGURE 7.**
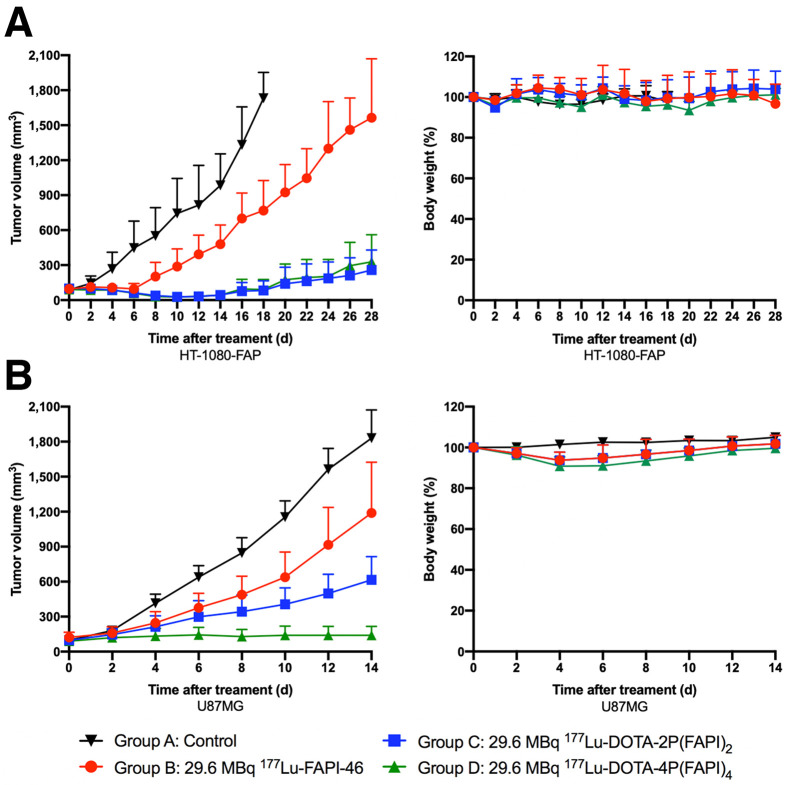
Radioligand therapy with ^177^Lu-DOTA-4P(FAPI)_4_, ^177^Lu-DOTA-2P(FAPI)_2_, and ^177^Lu-FAPI-46 in HT-1080-FAP and U87MG tumor–bearing mice. (A) Tumor growth curves and weight changes after treatment in HT-1080-FAP tumors (6/group). (B) Tumor growth curves and weight changes after treatment in U87MG tumors (6/group).

In HT-1080-FAP tumor–bearing mice, rapid tumor growth was observed in groups A (control) and B (29.6 MBq of ^177^Lu-FAPI-46 therapy). All tumor-bearing mice (6/6) in group A and most mice (5/6) in group B were euthanized by days 18 and 28, respectively, because of excessive tumor volumes (Figs. 7A and B). In groups C (29.6 MBq of ^177^Lu-DOTA-2P[FAPI]_2_) and D (29.6 MBq of ^177^Lu-DOTA-4P[FAPI]_4_), significant inhibition of tumor growth was observed, and most tumors started to shrink from day 6 and remained small until days 12–14, after which tumor volumes increased (Fig. 7B). No systemic toxicity due to radioligand therapy, determined by monitoring the body weight of the mice, was observed in any of the 4 groups. To further evaluate the toxic effects, hematoxylin and eosin staining of the selected nontarget organs was performed, which revealed no differences between the control and radioligand therapy groups (Supplemental Fig. 9).

In U87MG tumor–bearing mice, tumors in the control and ^177^Lu-FAPI-46 therapy groups both demonstrated fast growth, and all mice (6/6) in the control group and half the mice (3/6) in the ^177^Lu-FAPI-46 therapy group were euthanized by day 14 because of excessive tumor volumes. Although a better antitumor efficacy was observed in the ^177^Lu-FAPI dimer group (median survival time not reached) than in the control group (median survival, 12 d) and the ^177^Lu-FAPI-46 group (median survival, 14 d), the ^177^Lu-FAPI tetramer (median survival time not reached) yielded the greatest inhibition of tumor growth among all 4 groups (Fig. 7). In brief, the tumor volume in ^177^Lu-FAPI tetramer group was significantly less than in the FAPI dimer, FAPI-46, and control groups at day 14 after treatment (140.28 ± 76.36 mm^3^ vs. 616.14 ± 198.2 mm^3^ vs. 1,189.16 ± 435.26 mm^3^ vs. 1,830.18 ± 242.25 mm^3^; all *P* < 0.001).

## DISCUSSION

In the past 3 y, many clinical studies have explored the potential of FAP-targeted radioligand therapy with ^177^Lu- or ^90^Y-labeled FAPIs (8*,*^9^). However, most have revealed unsatisfactory therapeutic responses, mainly because of fast blood clearance accompanied by relatively short tumor retention. Therefore, various strategies have been developed to prolong the in vivo half-life of radiolabeled FAPIs to improve tumor uptake and retention.

An important strategy to enhance tumor uptake and retention is to harness the polyvalency effect of multimerization, which has been used in the development of arginylglycylaspartic acid peptides to improve their pharmacokinetics (17). Recently, we applied the multivalency concept to develop a dimeric FAPI molecule, DOTA-2P(FAPI)_2_ (12), which demonstrated enhanced tumor uptake and retention properties for dimers compared with monomers in patient-derived xenografts and patients with cancer. On the basis of those results, we synthesized tetrameric FAPI molecules with 4 repeating FAPI-46 units connected by 4 mini-PEG spacers. We hypothesized that multimerization to tetrameric FAPIs would further improve their tumor accumulation and retention because of adequate contact with the FAP-binding pocket located in the extracellular segment of cancer-associated fibroblasts.

The high labeling yield, radiochemical purity, and stability of the FAPI tetramer indicate that it is a convenient precursor for radiolabeling and application. Subsequently, radioligand-binding assays were used to examine the FAP-binding affinity of FAPI tetramers, dimers, and monomers. However, comparable 50% inhibitory concentrations were observed for all 3 FAPI variants. Multimeric FAPI molecules are not necessarily multivalent. The key to bivalency and tetravalency is the distance between the binding motifs. In this study, a FAP-binding affinity of the FAPI tetramer and dimer comparable to that of FAPI-46 indicates that the distance between binding motifs in DOTA-4P(FAPI)_4_ and DOTA-2P(FAPI)_2_ may not be sufficiently long for them to achieve tetravalency or bivalency. In addition, the bivalency and tetravalency of multimeric FAPI molecules also depend on FAP density. If FAP density is low, the distance between neighboring FAP sites will be long, and it may be more difficult for multiple multimers to simultaneously bind to FAP binding sites.

The tetramer ^68^Ga-DOTA-4P(FAPI)_4_ exhibited prominent uptake in the FAP-transfected tumor xenograft HT-1080-FAP, and its excretion route was primarily through the kidneys. However, it exhibited a similar initial tumor uptake and slightly longer tumor retention than those of ^68^Ga-DOTA-2P(FAPI)_2_ and ^68^Ga-FAPI-46, as may be explained by the intense FAP expression in this special tumor xenograft. In another tumor xenograft, U87MG, the tumor uptake of ^68^Ga-DOTA-4P(FAPI)_4_ was significantly higher than that of ^68^Ga-DOTA-2P(FAPI)_2_ and ^68^Ga-FAPI-46. In the blocking study, the tumor uptake of ^68^Ga-DOTA-4P(FAPI)_4_ decreased significantly when the mice were injected with unlabeled FAPI-46 1 h after injection, suggesting that the high tumor uptake of ^68^Ga-DOTA-4P(FAPI)_4_ was primarily a factor of its excellent FAP-targeting ability in vivo.

However, the relatively short half-life of ^68^Ga limits the observation time of tumor retention. Therefore, the FAPI tetramer and dimer were labeled with ^64^Cu to further evaluate their in vivo characteristics. The tetramer ^64^Cu-NOTA-4P(FAPI)_4_ exhibited a slightly higher initial tumor uptake and longer retention than ^64^Cu-NOTA-2P(FAPI)_2_. Compared with the molecular size of the FAPI monomer and dimer, the larger size of the FAPI tetramer may explain its longer circulation time and slower tumor washout. In contrast, as the greater number of FAP binding sites on FAPI tetramers will increase the local concentration of other FAPI motifs in the vicinity of FAP sites, the locally increased FAPI concentration may explain the higher tumor uptake of radiolabeled FAPI tetramers and dimers than of their monomeric analogs (18). The higher liver uptake of ^64^Cu-labeled radiopharmaceuticals may be attributed to the dissociation of free copper ions from the radiopharmaceuticals in vivo (19*,*^20^), which was also observed in previous studies. The liver uptake of ^64^Cu-NOTA–arginylglycylaspartic acid–bombesin was relatively lower than that of other ^64^Cu-DOTA radiotracers but higher than that of ^68^Ga-NOTA–arginylglycylaspartic acid–bombesin, possibly because of the higher chelating ability of NOTA with ^68^Ga than of NOTA with ^64^Cu (21). However, other factors, such as radiotracer stability and metabolism, can also contribute to the increased liver uptake. Increased liver uptake of a ^64^Cu-NOTA agent was also reported in PEG2-RM26 studies, partly because of the transchelation of ^64^Cu^2+^ to the serum components or superoxide dismutase that can accumulate in the liver tissue (22). Further studies are needed to fully elucidate the mechanisms underlying the liver uptake of ^64^Cu-labeled radiotracers.

Compared with FAPI dimers and monomers, the FAPI tetramer exhibited significantly higher uptake in certain nontarget organs, especially the kidney and liver, as reflected by PET and SPECT imaging and biodistribution studies. The relatively high uptake of the FAPI tetramer by the kidneys may be explained by different mechanisms. First, we speculate that the increased renal uptake of the FAPI tetramer may be partially related to the 4 mini-PEG spacers. PEGylation is a strategy widely used to improve the in vivo pharmacokinetics of radiotracers, induce hydrophilicity, and increase kidney uptake (23). Additionally, the difference in charge between the 3 FAPI molecules may cause differences in tubular reabsorption, as reported in previous studies (24). Because of the presence of more guanidine groups, tetrameric FAPI is more positively charged than dimeric and monomeric FAPI. The larger molecular size of the FAPI tetramer could cause a longer circulation time and greater retention in the liver. The fact that the background of ^68^Ga-labeled FAPI tetramer was higher than that of the dimer and monomer may have had unfavorable effects on diagnostic application. However, the FAPI tetramer applied in our study was designed to improve tumor uptake and retention so as to enhance the antitumor efficacy of FAP-targeted radioligand therapy. Furthermore, FAPI monomers, such as FAPI-04 and FAPI-46, are excellent PET imaging agents for detecting FAP-positive lesions because of their favorable pharmacokinetics and high binding specificity to FAP.

The increased tumor uptake and prolonged tumor retention of DOTA-4P(FAPI)_4_ encouraged us to apply it in FAP-targeted radioligand therapy. As expected, a single dose of ^177^Lu-DOTA-4P(FAPI)_4_ demonstrated excellent antitumor ability in HT-1080-FAP tumor–bearing mice, whereas the tumors continued to grow in the control and ^177^Lu-FAPI-46 therapy groups. However, because HT-1080-FAP is a FAP-transfected tumor xenograft with extremely high FAP expression, both ^177^Lu-DOTA-4P(FAPI)_4_ and ^177^Lu-DOTA-2P(FAPI)_2_ rapidly eradicated the tumors, with no observed difference between them. Therefore, another tumor xenograft, U87MG, was used to evaluate the antitumor ability of ^177^Lu-DOTA-4P(FAPI)_4_. This human glioblastoma cell–derived xenograft adequately recruits mouse fibroblasts during tumor growth and has been reported as a FAP-positive tumor model (25). Impressively, ^177^Lu-DOTA-4P(FAPI)_4_ demonstrated significantly better antitumor efficacy than did ^177^Lu-DOTA-2P(FAPI)_2_ and ^177^Lu-FAPI-46, indicating potential for the use of radiolabeled FAPI tetramers as theranostic agents.

However, the multimerization strategy may be a double-edged sword in the development of radiopharmaceuticals. In addition to improved tumor uptake and retention, it results in higher radiotracer uptake in normal organs, particularly the kidneys and liver. The increased accumulation of radioactivity in normal organs may result in the delivery of unnecessary radiation doses, which may affect the future clinical translation of these molecules into viable treatments. Whether the positive effects of increased tumor uptake offset the potential side effects of increased liver and kidney uptake is unclear. Increased liver and kidney uptake may be undesirable; however, it may be an acceptable trade-off if the benefits of increased tumor uptake are significant. In tumors with high expression of FAP, such as HT-1080-FAP, radioligand therapy with a FAPI dimer may lead to similar antitumor efficacy but fewer side effects than for a FAPI tetramer. However, ^68^Ga PET imaging and ^177^Lu-radioligand therapy in U87MG tumor–bearing mice revealed that the tetramer itself acts as a double titer of the dimer, thereby improving its antitumor efficacy. Therefore, radioligand therapy with a FAPI tetramer may be more appropriate than that with a FAPI dimer in tumors with moderate or mild expression of FAP. The selection of the dimer or tetramer ultimately depends on the specific circumstances of the treatment objectives and the potential benefits and risks associated with each option. Therefore, appropriate modifications by changing the linker or chelator are needed to improve the pharmacokinetics of FAPI-based radiopharmaceuticals (25), especially to improve their FAP-targeting capabilities and reduce radiotracer accumulation in noncancerous organs.

## CONCLUSION

The radiolabeled FAPI tetramer exhibited higher accumulation and longer retention in the tumor than did its dimeric and monomeric counterparts. The improved pharmacologic properties of ^177^Lu-DOTA-4P(FAPI)_4_ resulted in excellent antitumor ability in HT-1080-FAP and U87MG tumor–bearing mice. The information obtained here may guide the future development of FAP-targeted imaging and radioligand therapy.

## DISCLOSURE

This work was funded by the National Natural Science Foundation of China (82071961, 82272037), the Fujian Research and Training Grants for Young and Middle-Aged Leaders in Healthcare, the Key Scientific Research Program for Yong Scholars in Fujian (2021ZQNZD016), the Fujian Natural Science Foundation for Distinguished Young Scholars (2022J01310623), and the Key Medical and Health Projects in Xiamen (3502Z20209002). Liang Zhao was partially funded by the China Scholarship Council. No other potential conflict of interest relevant to this article was reported.

**KEY POINTS**

**QUESTION:** Compared with FAPI monomers and dimers, do FAPI tetramers demonstrate enhanced tumor uptake, prolonged tumor retention, and an improved radioligand therapeutic ability?

**PERTINENT FINDINGS:** FAPI tetrameric radiopharmaceuticals exhibited significantly increased tumor uptake and retention compared with their monomeric and dimeric counterparts. The ^177^Lu-FAPI tetramer demonstrated remarkable inhibition of tumor growth in both HT-1080-FAP and U87MG tumors, with negligible side effects.

**IMPLICATIONS FOR PATIENT CARE:** The formation of FAPI tetramers via multimerization is a promising strategy in the development of FAP-targeted radiopharmaceuticals.
